# Microdialysis-Assessed Adipose Tissue Metabolism, Circulating Cytokines and Outcome in Critical Illness

**DOI:** 10.3390/metabo8040062

**Published:** 2018-10-06

**Authors:** Ioannis Ilias, Sofia Apollonatou, Nikitas Nikitas, Maria Theodorakopoulou, Alice G Vassiliou, Anastasia Kotanidou, Ioanna Dimopoulou

**Affiliations:** 1 Endocrine Unit, Elena Venizelou Hospital, GR-11521 Athens, Greece; 2 Second Department of Critical Care Medicine, Attikon University Hospital, National and Kapodistrian University of Athens, Medical School, GR-12462 Athens, Greece; sofapol@yahoo.gr (S.A.); Mariatheodor10@gmail.com (M.T.); 3 Department of Critical Care Medicine, North Middlesex Hospital, London N18 1QX, UK; Nikitas.nikitas@outlook.com; 4 First Department of Critical Care Medicine, Evangelismos Hospital, National and Kapodistrian University of Athens, Medical School, GR-10552 Athens, Greece; alvass75@gmail.com (A.G.V.); akotanid@med.uoa.gr (A.K.); idimo@otenet.gr (I.D.)

**Keywords:** intensive care unit, lactate clearance, microdialysis, tissue ischemia

## Abstract

Microdialysis (MD) can provide continuous information about tissue composition. To assess in critically ill patients adipose tissue metabolic patterns, the relationships between metabolic patterns and blood cytokine concentration associations of adipose tissue energy metabolism and clinical outcome we studied 203 mechanically ventilated general intensive care unit (ICU) patients. Upon ICU admission an MD catheter was inserted into the subcutaneous adipose tissue of the upper thigh to measure lactate (L), glucose, pyruvate (P), and glycerol. Serum concentrations of IL-10, IL-6, IL-8, and TNF-α were determined within 48 h from ICU admission. Mitochondrial dysfunction was defined as L/P ratio >30 and pyruvate ≥70 μmol/L, ischemia as L/P ratio >30 and pyruvate <70 μmol/L and no ischemia/no mitochondrial dysfunction (i.e., aerobic metabolism) was as L/P ratio ≤30. Metabolism was aerobic in 74% of patients. In 13% of patients there was biochemical evidence of ischemia and in 13% of patients of mitochondrial dysfunction. Mitochondrial dysfunction was associated with poor outcome. In conclusion, MD showed that about two thirds of critically ill patients have normal aerobic adipose tissue metabolism. Mitochondrial dysfunction was not common but was associated with poor outcome. Identifying subgroups of critically ill patients is crucial as different treatment strategies may improve survival.

## 1. Introduction

Microdialysis (MD) is a minimally invasive technique, easily used at the bedside; this technique can provide continuous information about interstitial tissue fluid/space composition. A solute is slowly infused into an interstitially placed catheter at a constant velocity. Fenestrated membranes at the tip of the catheter permit free diffusion of molecules between the interstitium and the perfusate, which is subsequently collected for analysis [1,2,3]. MD has been applied to the study of tissue chemistry in several human organs, although most experience has been acquired in the setting of neurocritical care. Routinely measured substances include glucose, lactate, glycerol, glutamate, and pyruvate—to calculate the lactate to pyruvate (L/P) ratio. Glucose is a surrogate for tissue ischemia and lactate provides a measure of oxygenation or a sign of hypermetabolism. The L/P ratio is a marker of changes in the redox state of cells, which in turn is determined by oxygen availability (ischemia/hypoxia) or by oxidative metabolism (mitochondrial dysfunction). Glycerol, abundant in cell membranes, serves as a biomarker of ongoing cell damage. Glutamate, the predominant excitatory neurotransmitter, can provoke cell damage (“excitotoxicity”), being thus, an indirect marker of cell decomposition [4,5]. Microdialysis currently is part of the multimodal monitoring in neurocritical care and is a useful tool in predicting outcome and guiding decision making [6].

A number of investigators have tried to find uses for MD in general intensive care unit (ICU) patients. Recently, others [7,8,9] and our group [10,11,12,13,14,15,16] have carried out MD studies in critically ill septic or non-septic patients that have enhanced our understanding of biochemical and pathophysiological processes. In sepsis, MD can detect tissue metabolic abnormalities that are incongruous with disease severity, clinical outcome [10], type of underlying infection or responsible pathogen [11]. In septic shock, the adipose tissue L/P ratio has a prognostic capability similar to that of the Acute Physiology and Chronic Health Evaluation (APACHE II) scale [12]. Of importance, some of the MD-assessed abnormalities are not reflected by conventional blood measurements [13]. Finally, the well-known association between lipolysis and cortisol has been verified, with MD, at the tissue level [14]. In critical illness, MD has also been used to monitor the efficacy of interventions in improving cellular metabolism. In this regard, the tissue L/P ratio has been found to decrease after red blood cell transfusions [15] or after the administration of inotropic/vasoactive agents [17].

Critical illness, in particular sepsis, triggers a complex cascade of events associated with alterations in inflammatory/immune/hormonal responses that are largely orchestrated by cytokines. The complex network of cytokine balance pro-inflammatory and anti-inflammatory effects and their uncontrolled production can result in organ failure and death [18]. However, not all cytokine functions are limited to the immune system, as they also affect the metabolic response to severe illness. At the onset, the systemic inflammatory response is accompanied by generalized hypermetabolism, a condition characterized by marked substrate mobilization and catabolism [19]. Adipose tissue plays an important regulatory role in metabolism not only by storing and liberating high-energy compounds according to requirements, but also by synthesizing a number of substances that regulate homeostasis [20].

We hypothesized—based on previous research works on patients with brain lesions, injuries and infections [21,22,23,24]—that mitochondrial dysfunction and ischemia would affect adipose tissue metabolism in 30%–70% of critically ill patients. Thus, we carried out this study in critically ill patients to investigate: (i) the type of adipose tissue metabolic patterns; (ii) the relationship between metabolic patterns and blood cytokine concentrations; and (iii) the association of adipose tissue energy metabolism and clinical outcome (ICU mortality). To this end, MD was implemented by inserting an MD catheter into subcutaneous adipose tissue in a large group of mechanically ventilated, septic and non-septic, medical and surgical ICU patients.

## 2. Results

In this study, we investigated 203 (126 males) critically ill patients, with a median age of 67 years (range: 18–92 years). They consisted of medical (*n* = 161) or surgical (*n* = 42) patients, all intubated and mechanically ventilated. Of the 203 patients, 179 (88%) were septic (septic shock, *n* = 133; sepsis, *n* = 46), whereas 24 had SIRS [25]. The most common infection was pneumonia followed by intra-abdominal infection. Other infections included endocarditis, meningitis–encephalitis, and neck abscess. A subset of patients (*n* = 36) had bacteraemia.

The patients were further classified biochemically according to the definitions given previously as follows: mitochondrial dysfunction (*n* = 27/203, 13%), ischemia (*n* = 27/203, 13%) and no ischemia/no mitochondrial dysfunction, i.e., aerobic metabolism (*n* = 149/203, 74%). Table 1 provides the main demographics and clinical characteristics, along with laboratory data of the three groups.

Variables are given at baseline (day 1), except the Sequential Organ Failure Assessment (SOFA) score, dose of noradrenaline (mean daily dose), and blood lactate, which are shown on days 1–3. The SOFA score tended to be higher in mitochondrial dysfunction compared to no ischemia/no mitochondrial dysfunction on days 1–3, but this reached statistical significance only on day 2. Patients with mitochondrial dysfunction had a higher heart rate, lower platelets, and lower PO_2_/FIO_2_ vs. no ischemia/no mitochondrial dysfunction. The mean daily dose of noradrenaline was higher in mitochondrial dysfunction vs. no ischemia/no mitochondrial dysfunction on days 1–3. The highest blood lactate was observed in mitochondrial dysfunction; it was significantly higher in mitochondrial dysfunction compared to both no ischemia/no mitochondrial dysfunction and to ischemia on days 1–3. Mitochondrial dysfunction was present during three days (in *n* = 12/27 patients, 44%), during one day (in *n* = 10/27 patients, 37%) and during two days (in *n* = 5/27 patients, 19%). Ischemia was present during two days (in *n* = 12/27 patients, 44%), during one day (in *n* = 9/27 patients, 33%) and during three days (in *n* = 6/27 patients, 22%). None of the patients had both mitochondrial dysfunction and ischemia.

Microdialysis-derived metabolites in the three groups on days 1–3 are shown in Table 2.

Given that patients’ classification was based on L/P ratios and pyruvate levels these variables are presented, but are not included in statistical comparisons. In mitochondrial dysfunction, median tissue lactate was elevated (>2 mmol/L) and was significantly higher compared to no ischemia/no mitochondrial dysfunction and to ischemia on days 1–3. Lactate was higher in patients with no ischemia/no mitochondrial dysfunction (the majority had aerobic hyperglycolysis as discussed below) compared to ischemia on days 1 and 2. Median tissue glucose was within normal limits in mitochondrial dysfunction and in ischemia/no mitochondrial dysfunction, however, it was low (<5 mmol/L) in ischemia. Glucose was significantly lower in ischemia compared to mitochondrial dysfunction and to no ischemia/no mitochondrial dysfunction on days 1–3. Tissue glycerol was significantly lower in ischemia compared to mitochondrial dysfunction on day 1, and to no ischemia/no mitochondrial dysfunction on days 1–3.

Overall, 148/203 (73%) patients had lipolysis, i.e., mean tissue glycerol was higher than 200 μmol/L during at least one of the three days. Lipolysis was equally present in all groups, including mitochondrial dysfunction (*n* = 17/27 or 63%), ischemia (*n* = 16/27 or 59%) and no ischemia/no mitochondrial dysfunction (*n* = 115/149 or 77%) (*p* = 0.55).

Of the 149 patients with no ischemia/no mitochondrial dysfunction, 97 patients (65%) had aerobic hyperglycolysis, defined as a L/P ratio ≤30 along with a tissue lactate >2 mmol/L.

Blood cytokines were measured in 117/203 patients (58%). In the patient population as a whole, median IL-6 was 29 pg/mL (range: 0.20–4782 pg/mL), median IL-8 was 31 pg/mL (range: 1.52–4746 pg/mL), median IL-10 was 29 pg/mL (range: 0.57–3373 pg/mL) and median TNF-α was 9 pg/mL (range: 0.20–249 pg/mL). IL-6 correlated with SOFA score on day 1 (*r* = 0.31, *p* = 0.0008), APACHE II (*r* = 0.32, *p* = 0.0004), blood lactate on day 1 (*r* = 0.36, *p* < 0.001), and mean dose of noradrenaline on day 1 (*r* = 0.23, *p* = 0.03). The L/P ratio on day 1 correlated with IL-6 (*r* = 0.25, *p* = 0.007), IL-8 (*r* = 0.23, *p* = 0.01), and IL-10 (*r* = 0.19, *p* = 0.04). There were no correlations between TNF-α and L/P ratio.

Blood cytokine levels in the three groups defined as mitochondrial dysfunction, ischemia, and no ischemia/no mitochondrial dysfunction are shown in Table 3.

All cytokines were higher in mitochondrial dysfunction. Patients with mitochondrial dysfunction had statistically significant higher IL-6 and IL-8 compared to those with no ischemia/no mitochondrial dysfunction; they also had higher IL-6 than patients with ischemia. In contrast, IL-10 and TNF-α were comparable in the three groups.

Patients who had lipolysis also had higher IL-8 (41 pg/mL vs. 28 pg/mL, *p* = 0.03), and IL-10 (20 pg/mL vs. 7 pg/mL, *p* = 0.002) compared to those without lipolysis. In addition, tissue glycerol on day 1 correlated with IL-6 (*r* = 0.25, *p* = 0.006), IL-8 (*r* = 0.28, *p* = 0.002), and IL-10 (*r* = 0.28, *p* = 0.002). There were no correlations between tissue glycerol levels and TNF-α.

Patients with aerobic hyperglycolysis had similar blood cytokine levels with those with no anaerobic glycolyis (Il-10 = 12 pg/mL vs. 16 pg/mL, *p* = 0.45; IL-6 = 24 pg/mL vs. 30 pg/mL, *p* = 0.32; IL-8 = 37 pg/mL vs. 31 pg/mL, *p* = 0.72; TNF-α = 9 pg/mL vs. 9 pg/mL, *p* = 0.82). However, tissue lactate on day 1 correlated both with IL-6 (*r* = 0.21, *p* = 0.02) and IL-8 (*r* = 0.20, *p* = 0.03). There were no correlations between tissue lactate and IL-10 or TNF-α.

In the entire patient population, 28-days mortality and ICU mortality were 37% and 54% respectively. Subgroup analysis showed that the 28-days mortality was higher in patients with mitochondrial dysfunction: 63% in mitochondrial dysfunction vs. 48% in patients with ischemia vs. 30% in patients with neither mitochondrial dysfunction nor ischemia (*p* = 0.002, between mitochondrial dysfunction vs. no mitochondrial dysfunction/no ischemia). Logistic regression analysis revealed that mitochondrial dysfunction was an independent predictor of poor outcome (28-days mortality) (O.R. = 3.73, 95% C.I. = 1.52–9.20, *p* = 0.004), in the presence of APACHE II score (O.R.=1.14, 95% = 1.07–1.21, *p* <0.001). Mortality in the ICU was also higher in patients with mitochondrial dysfunction: 77% with mitochondrial dysfunction vs. 52% with ischemia vs. 50% with no ischemia/no mitochondrial dysfunction (*p* = 0.02, between mitochondrial dysfunction vs. no mitochondrial dysfunction/no ischemia). Finally, patients with mitochondrial dysfunction died earlier in the ICU compared to those with no mitochondrial dysfunction/no ischemia (median: 5 days vs. 18 days, *p* = 0.001).

Non-survivors (28-days mortality) had higher blood cytokines’ levels compared to survivors: IL-6 = 92 pg/mL vs. 19 pg/mL, *p* <0.001; IL-8 = 66 pg/mL vs. 24 pg/mL, *p* <0.001; IL-10 = 30 pg/mL vs. 11 pg/mL, *p* <0.001; and TNF-α = 12 pg/mL vs. 6 pg/mL, *p* = 0.009.

## 3. Discussion

In the present study we investigated adipose tissue metabolic patterns in critically ill patients (88% with sepsis) by inserting an MD catheter in the subcutaneous adipose tissue of the upper thigh. We used the relation between L/P ratio and the pyruvate concentration to characterize metabolic patterns. The major findings can be summarized as follows: (i) in the vast majority of patients (74%) metabolism was aerobic (L/P ratio ≤30), with lipolysis (tissue glycerol >200 μmol/L) and hyperglycolysis (L/P ratio ≤30 and tissue lactate >2 mmol/L) being frequently observed (73% and 65% respectively); (ii) the rest of the patients (26%) had anaerobic metabolism, with biochemical evidence either of ischemia (13%) (L/P ratio >30 and pyruvate <70 μmol/L) or of mitochondrial dysfunction (13%) (L/P ratio >30 and pyruvate ≥70 μmol/L); (iii) at the tissue level, mitochondrial dysfunction was characterized by elevated lactate and ischemia by glucose nadirs; (iv) patients with mitochondrial dysfunction had a more pronounced systemic inflammatory response (i.e., blood IL-6 and IL-8 levels), and more severe organ dysfunction compared to those with aerobic metabolism; (v) ICU mortality was similar in mitochondrial dysfunction and ischemia, however, it was higher in the presence of mitochondrial dysfunction compared to the normal aerobic pattern. Finally, mitochondrial dysfunction was of an independent predictive value for poor outcome with the APACHE II score.

Adipose tissue has generally been viewed as an inert tissue that was a store for excess energy in the form of triglycerides (lipogenesis). Indeed, during times of metabolic stress, triglycerides are broken down and free fatty acids (FFAs) and glycerol are released into the circulation (lipolysis). FFAs are carried to the liver, muscles, and other tissues for oxidation, are converted into acetyl coenzyme A molecules and are used as an energy source by other tissues. Glycerol is carried to the liver for oxidation or gluconeogenesis. Balance usually exists between these two states, but dysregulation can occur, leading to either an excessive storage of lipid within adipose tissue or an excessive depletion in states of catabolism [20]. On the other hand, in the past two decades, adipose tissue has become recognized as a dynamic tissue involved in the regulation of metabolism, the inflammatory response, coagulation, and insulin sensitivity, all of which are major elements in the response to critical illness, particularly to sepsis [3,26].

The interstitial space is the “crossroad” of all substances passing between cells and blood capillaries. By monitoring by the bedside this compartment it is possible to get crucial information on energy metabolism at the tissue of interest [27]. In the present study, we placed an MD catheter in subcutaneous adipose tissue to study metabolic energy patterns, i.e., the redox state (L/P ratio), lipolysis (adipose tissue glycerol) and carbohydrate metabolism (adipose tissue glucose and lactate) in critically ill patients. Tissue lactate, pyruvate, and glycerol were measured, while the L/P ratio was calculated (the latter is a widely accepted marker of changes in the redox state of cells [1,2,4,5,6]). Tissue metabolic failure may be mediated by inadequate perfusion/oxygen delivery (ischemia), but also by a diminished capability of cells to use the available O_2_ as a consequence of mitochondrial damage and disruption in the electron transport chain (primary mitochondrial dysfunction). During ischemia, tissue supply of oxygen and substrates are diminished; the L/P ratio increases due to an increase in lactate and a decrease in pyruvate, and glucose is low. During mitochondrial dysfunction, blood supply, and delivery of substrates continues; thus, the increase in L/P ratio is characterized by very high lactate level and pyruvate concentration within or above normal limits, whereas tissue glucose remains within normal levels [28,29,30].

Based on previous clinical investigations [21,22,23,24] we defined an L/P ratio of 30 as the upper limit for normal aerobic metabolism. Our results show that the vast majority (149/203 patients or 74%) of critically ill, mechanically ventilated patients had biochemical evidence of aerobic adipose tissue metabolism, with L/P ratios being below 30 for the entire three-day observation period. Of the 203 patients, 148 patients (73%) had lipolysis (defined as tissue glycerol >200 μmol/L). As previously shown, increased lipolysis supports the concept of hypermetabolism in critical illness, in particular sepsis, in an effort to cope with increased metabolic demands [31]. In addition, 97 of the 149 patients with aerobic metabolism (65%) had hyperglycolysis (defined as a L/P ratio ≤30 along with a tissue lactate >2 mmol/L) [32], and this explains why tissue lactate was relatively high in this particular group, and even higher than in ischemia patients (see Table 2). Increased glycolysis is a hallmark of critical illness-related hypermetabolism, because oxidative phosphorylation runs at near maximal capacity, and, consequently, an increased energy demand should be applied by an increase in glycolysis [31]. In the present study, we defined ischemia as a L/P ratio >30 simultaneously with pyruvate <70 μmol/L, and mitochondrial dysfunction as a L/P ratio >30 at a pyruvate ≥70 μmol/L. Such criteria have been used in MD studies on brain-injured patients, including middle cerebral artery infarction, aneurysmal subarachnoid hemorrhage, bacterial meningitis, or trauma [21,22,23,24]. For the L/P ratio the upper normal level was set at 30 (normal mean + 2SD) and the lower normal level for pyruvate was set at 70 (normal mean – 2SD) [27,28,29,30]. We found that of the 203 patients studied, 27 patients (13%) had biochemical evidence of ischemia, which was in parallel with low tissue glucose (<5 mmol/L). As stated before, low tissue glucose is a hallmark of ischemia because the delivery of substrates is impaired. Of interest, similar results have been shown in patients with severe traumatic brain injury [24]. We also found that another 27 patients (13%) had evidence of mitochondrial dysfunction. Patients with mitochondrial dysfunction had higher blood lactate levels compared to those with ischemia and to those with aerobic metabolism. Per definition they had high tissue L/P ratios and normal-to-high pyruvate levels. Tissue lactate was higher in mitochondrial dysfunction compared to ischemia and to aerobic metabolism. Tissue glucose was within normal limits (about 5 mmol/L), indicating that the supply of glucose was sufficient to meet demands of the tissue. In the current study, we placed the MD catheter in subcutaneous adipose tissue; thus, tissue glycerol, which was normal-to-high in all groups, could not serve as a marker of cell membrane degradation as in brain injury, but reflected ongoing lipolysis [28].

Multiple organ failure (MOF) in the context of sepsis or acute illness is associated with overwhelming systemic inflammation with immune, metabolic, endocrine, and cardiovascular dysfunction [33]. Although a large variety of mediators have been implicated, a rapidly growing body of evidence suggests that proteins belonging to the cytokine family are decisive factors in determining the pathobiology of critical illness, particularly of sepsis [18,33]. In our study, we measured blood pro- and anti-inflammatory cytokines (i.e., IL-10, IL-6, IL-8, and TNF-α) in a significant subset of our patient population (117/203 patients or 58%) within 48 h from ICU admission. The majority of our patients were septic (88%). In accordance with previous findings, significant associations were observed between cytokine levels, severity of critical illness (i.e., APACHE II), degree of organ dysfunction (i.e., SOFA score), and clinical outcome [18,34,35]. Of note, our data showed that blood cytokine concentrations were related to MD-derived metabolites and to metabolic patterns; certain cytokines correlated positively with L/P ratio, tissue lactate along with tissue glycerol, indicating that cytokines are related to the redox state of the cells, lipolysis, and hyperglucolysis. The highest cytokine concentrations were observed in mitochondrial dysfunction and, in particular, IL-6, a potent pro-inflammatory cytokine, was significantly higher compared to both aerobic metabolism and ischemia. In our study, mitochondrial dysfunction was related to greater organ dysfunction and to poor clinical outcome within the ICU. It is generally accepted, that in human sepsis, except macro- and microcirculatory hemodynamic disturbances, mitochondrial dysfunction associated with the production of pro-inflammatory mediators may occur. Under these circumstances, impairment of mitochondrial function significantly contributes to multiple organ failure and death [36].

Limitations of the current study should be acknowledged. The study population was heterogeneous (with medical and surgical patients) and the patients were classified using criteria applied in brain injury (although the L/P ratio and pyruvate are considered to be essentially constant in all tissues [28,37]). In the brain MD studies [21,22,23,24] the catheters were placed directly in the affected tissue (namely cerebral tissue), while in our study the MD catheters were placed in adipose tissue (with the aim of studying adipose tissue metabolism). The cytokines that we assessed were measured in blood and not at the subcutaneous adipose tissue level (this would have required insertion of a second large-pore MD catheter, permitting substances with higher molecular weight to be collected and analyzed).

## 4. Materials and Methods

### 4.1. Study Population

In this retrospective, observational study, we investigated medical and surgical mechanically ventilated patients hospitalized in the adult ICU of “Attikon” Hospital over a 5-year period. Patients analyzed in the current study are partly shared with other publications by our research group [10,11,12,13,14,15,16]. Patients were not enrolled in the study if one of the following exclusion criteria was present: age less than 18 years; mechanical ventilation for more than 48 h before ICU admission; no need for intubation and mechanical ventilation during ICU stay; do-not-resuscitate clinical conditions; brain-death upon ICU entry; and HIV infection. The following data were recorded for each patient upon study entry: age, gender, and admission diagnosis (i.e., medical or surgical patients). Disease severity was assessed according to Acute Physiology and Chronic Health Evaluation (APACHE II) score [38]. The degree of organ dysfunction was quantified by the Sequential Organ Failure Assessment (SOFA) score every day throughout the study period (days 1–3) [39]. Finally, 28-days mortality and ICU mortality were also recorded. The study was approved by the hospital’s Ethics Committee and informed consent was obtained from the patients’ relatives.

### 4.2. Adipose Tissue/Blood Sample Collection and Measurements

Upon ICU admission (day 1) an MD catheter (CMA 60, CMA Microdialysis AB, Stockholm, Sweden) was inserted under sterile conditions into the subcutaneous adipose tissue of the upper thigh. The length of the dialysis membrane at the distal end of the catheter was 30 mm. The cut-off value of this membrane was 20.000 Daltons. The catheter was continuously perfused with a lactate-free Ringer’s solution (Perfusion fluid T1, CMA Microdialysis AB, Stockholm, Sweden; Na^+^, 147 mM; K^+^, 4 mM; Ca^2+^, 2.3 mM; Cl^−^, 156 mM; pH 6) and was pumped at a speed of 0.3 μL/min using a CMA 106 pump (CMA Microdialysis AB, Stockholm, Sweden). The length of the MD membrane and the slow perfusion flow rate guarantee a high recovery rate for molecules up to 20.000 Daltons, providing thus true tissue concentrations [1,2]. The first dialysate samples were collected in microvials at least two hours after the insertion of the catheter to avoid the effect of placement trauma on metabolite measurements. Samples were analyzed immediately for lactate glucose, pyruvate, and glycerol levels by a mobile, fully automated analyzer (CMA 600 Microdialysis Analyzer, CMA Microdialysis AB, Stockholm, Sweden) and the L/P ratio was calculated. Sampling was performed 6 times per day. The exact sampling time-intervals during the day were the following: (1) 05:00–09:00; (2) 09:00–13:00; (3) 13:00–17:00; (4) 17:00–21:00; (5) 21:00–01: 00; (6) 01:00–05:00. The daily mean values of all MD-derived metabolites were calculated for each patient. In the present study, measurements of the three first days (days 1–3) were considered for analysis. In general, normal values for metabolites are as follows: glucose 1.5–2 mmol/L (brain), 5 mmol/L (adipose tissue); lactate 2 mmol/L; pyruvate 120 μmol/L; L/P ratio 15–20; and glycerol 50 μmol/L (brain), 200 μmol/L (adipose tissue) [24].

Among microdialysis-derived metabolites, L/P ratio and pyruvate were used to classify patients into three groups: those with mitochondrial dysfunction, those with ischemia, and those with no ischemia/no mitochondrial dysfunction (i.e., presenting aerobic metabolism). This classification was based on previous clinical studies in brain injury [27,28,29,30]. Mitochondrial dysfunction was defined as an L/P ratio >30 at a pyruvate ≥70 μmol/L. Ischemia was defined as a L/P ratio >30 simultaneously with pyruvate <70 μmol/L. No ischemia/no mitochondrial dysfunction (i.e., aerobic metabolism) was defined as a L/P ratio ≤30. Data from patients were included into the three diagnostic groups when these criteria were fulfilled during at least in one of the three consecutive days. Thus, the no ischemia/no mitochondrial dysfunction group consisted of patients having a L/P ratio ≤30 during the entire three-day study period. In the group of no ischemia/no mitochondrial dysfunction, aerobic hyperglycolysis was defined as tissue lactate >2 mmol/L [32]. Given that the MD catheter was inserted in adipose tissue, an increase in tissue glycerol (>200 μmol/L) during at least one of the three consecutive days was interpreted as an indication of ongoing lipolysis [24].

Serum concentrations of IL-10, IL-6, IL-8, and TNF-α were determined within 48 h from ICU admission with Multiplex Human Th17 Magnetic Bead Panel (cat. HTH17MAG-14K) (Millipore Corporation, Billerica; normal levels set at ≤2.8 pg/mL for IL-10, ≤23.4 pg/mL for IL-6, ≤43.4 pg/mL IL-8, and ≤72 pg/mL for TNF-α [40]). None of the previous studies published by our group included cytokine measurements [10,11,12,13,14,15].

Routine hematological and biochemistry tests were collected on admission in the ICU. Concomitantly with dialysate sampling, arterial blood was drawn and analyzed for lactate on days 1–3 (GEM Premier 3000, Instrumentation Laboratory, Milan, Italy).

### 4.3. Statistical Analysis

All data were tested for normal distribution by the Kolmogorov–Smirnov test. Results are presented as means ± SD or medians and corresponding ranges. Kruskal–Wallis One Way Analysis of Variance on Ranks was used to compare values between the three groups (i.e., mitochondrial dysfunction, ischemia, no ischemia/no mitochondrial dysfunction). All pairwise multiple comparisons were performed by Dunn’s Method. The chi-square test was used for the respective comparisons in categorical variables within the three groups. Multiple logistic regression was used to assess the ability of the metabolic pattern having the highest mortality to predict 28-days mortality in the presence of APACHE II score. Odds ratios (O.R.) and 95% confidence intervals (C.I.) are reported. Spearman correlation coefficient (r_s_) assessed the associations between variables. In all analyses a *p* < 0.05 was considered statistically significant. Analyses were performed with SPSS 15.0 (SPSS Inc., Chicago, IL, USA) and SigmaPlot 11.0 software (Systat, San Jose, CA, USA).

## 5. Conclusions

Bedside MD showed that about two thirds of critically ill patients have normal aerobic adipose tissue metabolism. An adipose tissue metabolic/biochemical pattern interpreted as mitochondrial dysfunction (13%) or as ischemia (13%) was not common. Nevertheless, our results (with 26% in total of critically ill subjects showing mitochondrial dysfunction or ischemia in adipose tissue) are not at great variance from the ones obtained from brain and other tissue studies [21,22,23,24]; thus we believe that our hypothesis has not been refuted. Furthermore, mitochondrial dysfunction was associated with an enhanced inflammatory response, more profound organ dysfunction, and poor ICU outcome. Despite advances, mortality from critical illness remains high, and therefore, identifying subgroups of patients is crucial as different treatment strategies may improve patients’ survival.

## Figures and Tables

**Table 1:** Clinical characteristics and laboratory data in groups defined as mitochondrial dysfunction, ischemia, and no ischemia/no mitochondrial dysfunction.

		Mitochondrial Dysfunction (*n* = 27)	Ischemia (*n* = 27)	No ischemia/No Mitochondrial Dysfunction (*n* = 149)	*p*
Age (years) *		69	65	67	0.72
Gender (male/female), *n*		18/9	16/11	92/87	0.17
Medical/surgical patients, *n*		21/6	21/6	119/30	0.75
APACHE II score *		20	20	18	0.33
SOFA score *	d1	9	8	8	0.06
	d2	9	7	7	0.02 ^a^
	d3	9	6	7	0.09
Sepsis, *n* (%)		27 (100%)	25 (93%)	142 (97%)	0.75
Septic shock, *n* (%)		10 (37%)	9 (33%)	35 (23%)	0.31
Heart rate (beats/min) *		100	92	92	0.02 ^a^
Mean arterial pressure (mmHg) *		78	77	76	0.96
Temperature (°C) *		37.0	36.8	36.5	0.18
Hemoglobin (g/L) *		9.5	9.7	10.0	0.51
White blood cell count (× 10^3^/μL) *		13.020	10.446	12.355	0.79
Platelet count (× 10^6^/μL ) *		70.000	145.000	166.000	0.02 ^a^
Creatinine (mg/dL) *		1.8	1.2	1.3	0.16
Bilirubin (mg/dL) *		1.2	0.8	0.8	0.19
PO_2_/FIO_2_ *		140	165	220	<0.001 ^a^
C-reactive protein (mg/L) *		130	109	124	0.84
Noradrenaline dose (μcg/kg/min) *	d1	12	9	4	<0.001 ^a^
	d2	13	8	3	0.004 ^a^
	d3	5	3	2	0.017 ^a^
Blood lactate (mmol/L) *	d1	2.2	1.3	1.3	0.006 ^a,b^
	d2	2.0	1.2	1.2	0.007 ^a,b^
	d3	1.6	1.1	1.1	0.011 ^a,b^

* median values; ^a^ mitochondrial dysfunction vs. no ischemia/no mitochondrial dysfunction; ^b^ mitochondrial dysfunction vs. ischemia.

**Table 2:** Microdialysis (MD)-derived metabolites (median values) in the three groups defined as mitochondrial dysfunction, ischemia, and no ischemia/no mitochondrial dysfunction on days (d) 1–3.

		Mitochondrial Dysfunction (*n* = 27)	Ischemia (*n* = 27)	No Ischemia/No Mitochondrial Dysfunction (*n* = 149)	*p*
MD L/P ratio	d1	32	36	17	NP
	d2	43	39	17	NP
	d3	35	33	16	NP
MD Pyruvate (μmol/L)	d1	210	42	145	NP
	d2	149	35	124	NP
	d3	127	34	113	NP
MD Lactate (mmol/L)	d1	8.4	1.4	2.5	<0.001 ^a,b,c^
	d2	6.3	1.2	2.1	<0.001 ^a,b,c^
	d3	5.7	1.3	1.8	<0.001 ^a,b^
MD Glucose (mmol/L)	d1	4.3	2.0	5.6	<0.001 ^b,c^
	d2	5.5	2.0	6.7	<0.001 ^b,c^
	d3	6.0	2.4	6.1	<0.001 ^b,c^
MD Glycerol (μmol/L)	d1	313	127	237	0.009 ^b,c^
	d2	265	239	266	0.03 ^b^
	d3	205	205	272	0.02 ^b^

L/P = lactate to pyruvate; NP = not performed; ^a^ mitochondrial dysfunction vs. no ischemia/no mitochondrial dysfunction; ^b^ mitochondrial dysfunction vs. ischemia; ^c^ ischemia vs. no ischemia/mitochondrial dysfunction.

**Table 3:** Blood cytokine levels (median values) in the three groups defined as mitochondrial dysfunction, ischemia, and no ischemia/no mitochondrial dysfunction.

	Mitochondrial Dysfunction (*n* = 20)	Ischemia (*n* = 25)	No Ischemia/No Mitochondrial Dysfunction (*n* = 72)	*p* Value
IL-10 (pg/mL)	39	16	12	0.16
IL-6 (pg/mL)	330	23	23	0.003 ^a,b^
IL-8 (pg/mL)	92	30	27	0.03 ^a^
TNF-α (pg/mL)	17	9	8	0.43

IL = interleukin; TNF = tumor necrosis factor; ^a^ mitochondrial dysfunction vs. no ischemia/no mitochondrial dysfunction; ^b^ mitochondrial dysfunction vs. ischemia.
